# Supplementary material for: Preparation and Characterization of TiO2-PVDF/PMMA Blend Membranes Using an Alternative Non-Toxic Solvent for UF/MF and Photocatalytic Application
Source: Molecules. 2019 Feb 17;24(4):724. doi: 10.3390/molecules24040724 (PMC6412202; doi:10.3390/molecules24040724)
Supplement: Supplementary file 1 [file molecules-24-00724-s001.pdf]

Supporting material

Preparation and characterization of TiO_2-PVDF/PMMA blend membranes using an alternative non-toxic solvent for UF/MF and photocatalytic application

Ouassila Benhabiles [1,4], Francesco Galiano [2*], Tiziana Marino [2], Hacene Mahmoudi [3], Hakim Lounici [4,5], Alberto Figoli [2*]

[1] Unité de Développement des Equipements Solaires, UDES /Centre de Développement des Energies Renouvelables, CDER, *42004, Tipaza, Algeria.*
[2] Institute on Membrane Technology (ITM-CNR), Via P. Bucci 17c, 87036 Rende (CS), Italy
[3] Faculty of Technology, University Hassiba Benbouali of Chlef, Chlef, Algeria,
[4] Département génie de l'Environnement, Ecole Nationale Polytechnique, Alger, Algérie,
[5] Faculty of Science, University of Bouira, Algeria
* Correspondence: f.galiano@itm.cnr.it; a.figoli@itm.cnr.it;

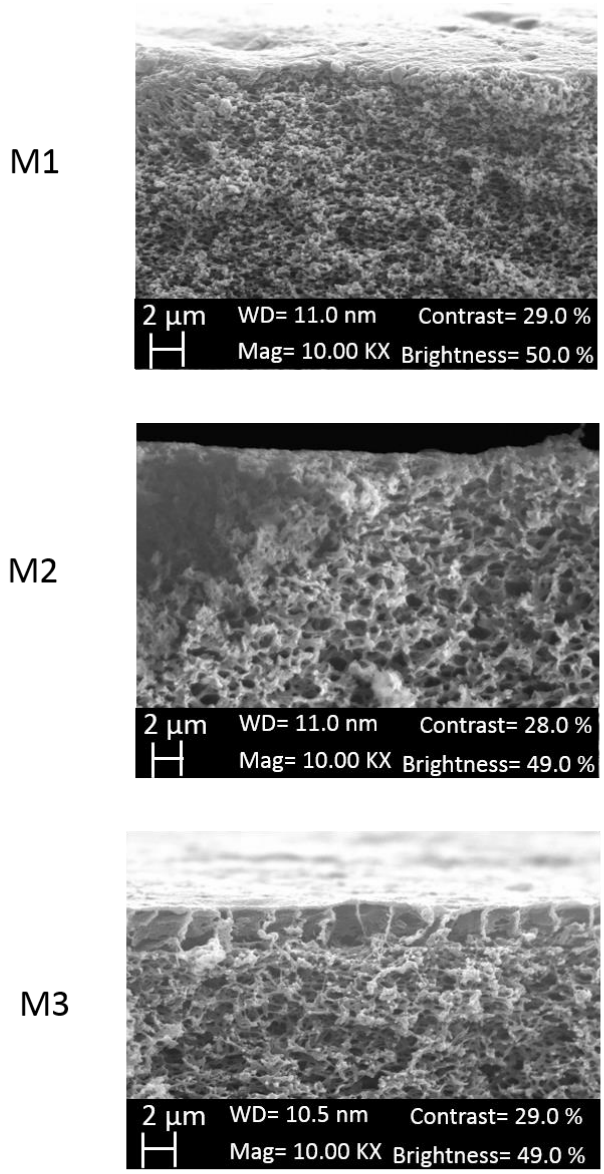

Figure S1. SEM magnification cross-sections of M1, M2 and M3 membranes

M4

M2

M5

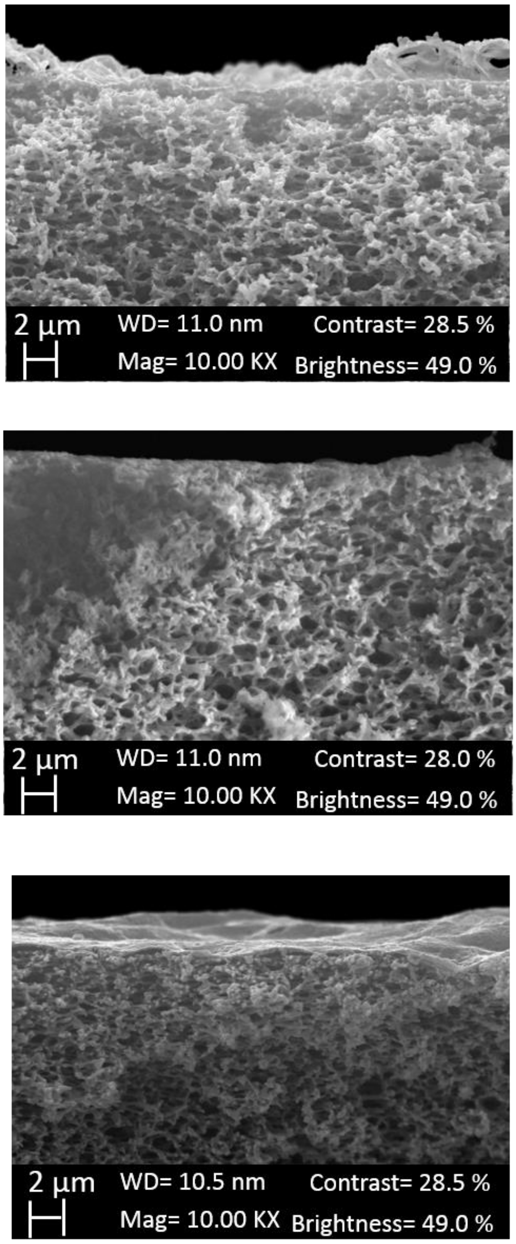

Figure S2. SEM magnification cross-sections of M4, M2 and M5 membranes

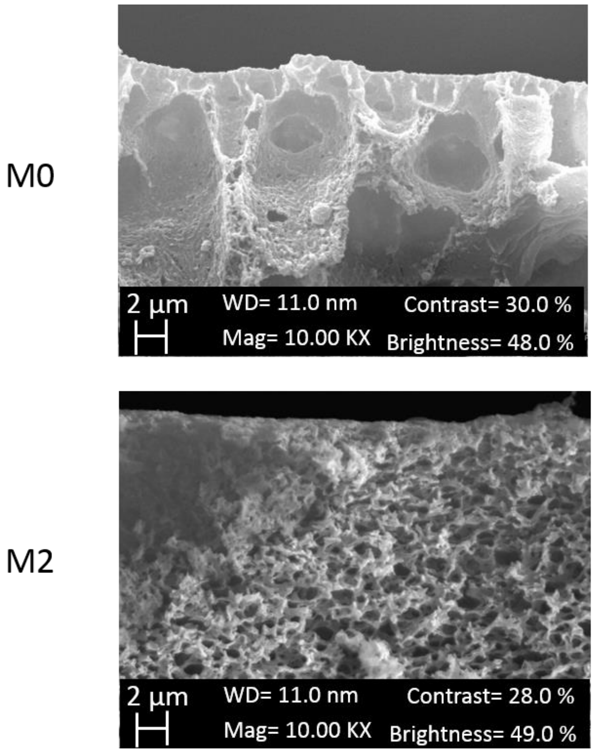

Figure S3. SEM magnification cross-sections of M0 and M2 and M5 membranes

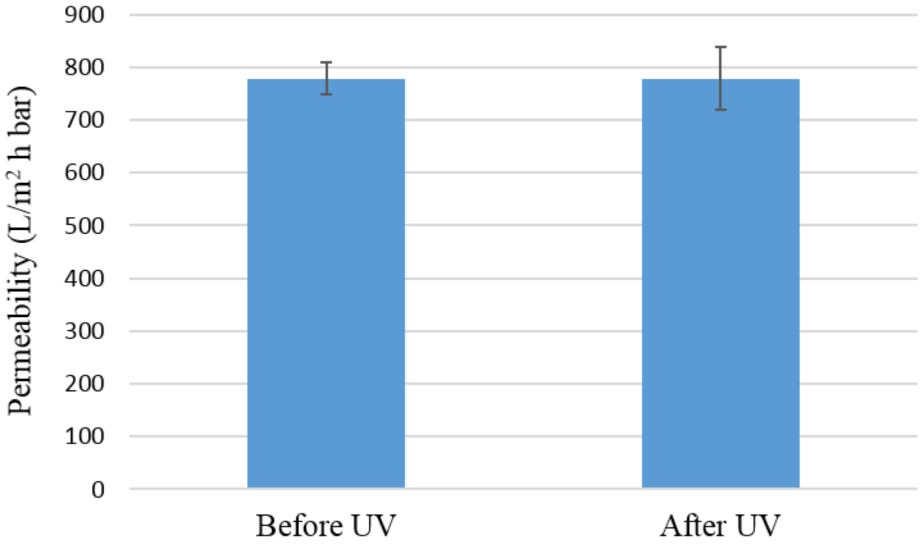

Figure S4. PWP of M8 membrane before and after UV irradiation
